# Modified electron beam output calibration based on IAEA Technical Report Series 398

**DOI:** 10.1002/acm2.13573

**Published:** 2022-02-28

**Authors:** Supriyanto Ardjo Pawiro, Dwi Aprilia Mahfirotin, Muhamad Iqbal Assegab, Wahyu Edy Wibowo

**Affiliations:** ^1^ Department of Physics Faculty of Mathematics and Natural Sciences Universitas Indonesia Depok West Java Indonesia; ^2^ Department of Radiation Oncology Dr. Cipto Mangunkusumo National General Hospital Jakarta Indonesia

**Keywords:** AAPM TG‐51 protocol, absorbed dose to water, electron beam, IAEA TRS‐398 protocol, modified calibration

## Abstract

**Purpose:**

The recently worldwide standard measurement of electron beam reference dosimetry include the International Atomic Energy Agency (IAEA) Technical Report Series (TRS)‐398 and Association of Physicists in Medicine (AAPM) Task Group (TG)‐51 protocols. Muir et al. have modified calibration methods for electron beam calibration based on AAPM TG‐51. They found that the use of cylindrical chambers at low energy gave acceptable results. In this study, we propose and report a modified calibration for electron beam based on IAEA TRS‐398, the standard reference dosimetry protocol worldwide.

**Methods:**

This work was carried out with energies of 6, 8, 10, 12, and 15 MeV. The electron beam is generated from Elektra Synergy Platform and Versa HD linear accelerator. The charge readings were measured with PTW 30013, IBA CC13, Exradin A1Sl, and Exradin A11 chambers connected to the electrometer. The dose calculation uses an equation of modified calibration for electron beam using the updated $k_{Q}$ factor in previous work. The absorbed dose to water for electron beam is expressed in dose per monitor unit (cGy/MU). Thus, we compared dose per monitor unit (*D*/MU) calculation using a modified calibration to TRS‐398.

**Results:**

In this work, we have succeeded in implementing the modified calibration of electron beam based on TRS‐398 by applying a cylindrical chamber in all energy beams and using the updated $k_{Q}$ factor. The ratio of the absorbed dose to water between original and modified calibration protocols of TRS‐398 (*D*_w_) for the cylindrical chamber was 1.002 on the Elekta Synergy Platform and 1.000 on the Versa HD while for the parallel‐plate chamber it was 1.013 on the Elekta Synergy Platform and 1.014 on the Versa HD. Based on these results, both the cylindrical and parallel‐plate chambers are still within the tolerance limit allowed by the TRS‐398 protocol, which is ±2%. Therefore, modified calibration based on TRS‐398 gives acceptable results and is simpler to use clinically.

## 1 INTRODUCTION

The International Atomic Energy Agency (IAEA) and the Association of Physicists in Medicine (AAPM) have issued several dosimetry protocols to calibrate high‐energy photons and electrons. Several dosimetry protocols have been issued IAEA Technical Report Series (TRS)‐277 (1987), IAEA TRS‐381 (1997), AAPM Task Group (TG)‐ 21 (1983), and AAPM TG‐39 (1994), are based on air kerma (exposure) and $N_{D,\mathrm{air}}$ to determine the absorbed dose to water.^1^ These protocols incorporated advances in radiation dosimetry that existed at that time, intending to increase photon and electron beam calibration accuracy.

In recent years, the main emphasis in standard laboratories worldwide has shifted from being based initially on exposure or air kerma to an absorbed dose to water.^2^ The absorbed dose to water is the principal fundamental quantity in radiotherapy because this quantity is closely related to the biological effects of radiation.^3^ The protocols that have been issued following the development of standards of absorbed dose to water include AAPM TG‐51 or TG‐51 protocol and IAEA TRS‐398 or TRS‐398 protocol. TG‐51 protocol is a new dosimetry protocol that has been developed and used predominantly in North America, which is based on the use of an ionization chamber calibrated in terms of absorbed dose to water in a Co‐60 gamma‐ray beam. TRS‐398 protocol is an International protocol as a code of practice for determining the absorbed dose of external radiation based on absorbed dose to water. These protocols are predominantly used throughout the rest of the world.

The equation of electron beam reference dosimetry in the TRS‐398 protocol requires a calibration factor of $N_{D,w,Qo\;}$ and a beam quality factor of $k_{Q,Q_{o}\;}.$ The TG‐51 protocol also requires a beam quality conversion factor which is denoted as $k_{Q}$. However, determining the beam quality factor $k_{Q}$ in electron beam is more complex. In an electron beam, the beam quality factor $k_{Q}$ is the product of three components, namely $k_{Q}=\;P_{\mathrm{gr}}^{Q}\;k_{R_{50}}^{\prime }k_{\mathrm{ecal}\;}$. Notation of $P_{\mathrm{gr}}^{Q}$ is only needed on cylindrical ionization chamber to correct for gradient effects at reference depths.^4^

Electron beam dosimetry based on TRS‐398 and TG‐51 protocols recommends using a parallel‐plate chamber in the electron beam, especially at low energies. A parallel‐plate chamber should be used at energy less than 6 MeV ($R_{50}\leq 2.6\;\mathrm{cm}$), and their use is recommended at beams of energy less than 10 MeV ($R_{50}\leq 4.3\;\mathrm{cm}$).^3^, ^5^, ^6^ This is because the use of a cylindrical chamber at low energy provides a fluency perturbation correction factor of up to 5%.^7^ However, the stability of the parallel‐plate chamber for measuring electron beam dosimetry at low energies (*E*_O_ < 10 MeV) raises questions.^8^ This is because the characteristics of the parallel‐plate chamber have the potential to cause problems. A current Monte Carlo calculation^9^ showed that the use of a parallel‐plate chamber resulted in a correction of up to 1.7% in NACP‐02 chamber and an even greater effect in water, especially at low‐energy electrons. The previous work^10^ also showed in their publication that the $k_{Q}$ factor for parallel‐plate chamber does not vary as previously assumed. The long‐term stability of the parallel‐plate chamber is also not as good as that of the cylindrical chamber which suggests that a cross‐calibration procedure is still preferred to use parallel‐plate chambers for reference dosimetry measurements.^11^ Therefore, the use of a stable cylindrical chamber would be more appropriate for measuring electron beam references even at low energies. The measurement by Muir and McEwen^11^ also showed that the chamber's perturbation correction in using a cylindrical chamber for electron beam dosimetry is independent of energy. In addition, the variability of relative ion chamber perturbation correction of the ionization chamber on the cylindrical chamber yields a value of less than 0.4%, which is not worse than the parallel‐plate chamber.^11^ Furthermore, there is a measurement work by Muir^12^ of modified calibration for electron beam calibration based on AAPM TG‐51. He found that the use of cylindrical chambers at low energy gave acceptable results. He also proposed the cylindrical chamber be appropriate for all‐electron beam energies to be used as reference dosimetry.

The measurement of modified calibration for electron beam by Muir^12^ used the updated $k_{Q}$ factor carried out by Muir and Rogers.^13^ The $k_{Q}$ factor was obtained using a Monte Carlo simulation with the EGSnrc system code, where this Monte Carlo simulation has included an implicit gradient correction factor $P_{\mathrm{gr}}^{Q}$.^13^ The equation of modified calibration for electron beam in recent work by Muir^12^ omitting the gradient correction factor $P_{\mathrm{gr}}^{Q}$ and replacing the notation $R_{50}$ to Q to emphasize that the gradient correction factor is implicitly taken into account. From the results measured by Muir,^12^ an acceptable dose measurement result was obtained when using a cylindrical chamber at all energies, even at low energy, without calculating the gradient correction factor.

This paper proposes a modified calibration for electron beam based on IAEA TRS‐398, the standard reference dosimetry protocol worldwide. Then, this study also reports the implementation of the modified calibration for electron beams in our institution based on IAEA TRS‐398. In this work, the output electron beam from two different types of linear accelerator Elekta is presented. The chambers used are PTW 30013, IBA CC13, Exradin A1Sl, and Exradin A11. The measurement results of the modified calibration for electron beams show good agreement with calibration based on TRS‐398. This will be very useful for clinical procedures for patients in our radiotherapy institution.

## 2 METHODS

### 2.1 Material

The absorbed dose to water dosimetry measurement was performed using Elekta Synergy Platform linear accelerator and Elekta Versa HD linear accelerator using electron beams with 6, 8, 10, 12, and 15 MeV. Three cylindrical‐type ionization chambers and a parallel‐plate chamber were employed in this work. The absorbed dose to water calibration factor $N_{D,w}^{\mathrm{Co}-60}$ for the four chambers was provided by the Indonesian secondary standard dosimetry laboratory (National Nuclear Energy Agency of Indonesia). The calibration factor $N_{D,w}^{\mathrm{Co}-60}$ for the PTW 30013, IBA CC13, Exradin A1Sl, and Exradin A11 chambers were 0.05389, 0.271, 0.6033, and 0.05026 Gy/nC, respectively. IBA CC13 and Exradin A11 were connected to a Max 4000 electrometer. Moreover, PTW 30013 and Exradin A1Sl were connected to a PTW Unidos and a Tomo electrometer.

Dosimetry for the absorbed dose to water and measurements of beam quality was performed using Blue water phantom (IBA Dosimetry) for the IBA CC13, Exradin A1Sl, and Exradin A11, and using a 30 × 30 × 30 cm^3^ water phantom for PTW 30013. A 10 × 10 cm^2^ clinical applicator was used to shape the field. Chambers were irradiated to 100 MU. The operating voltage for the PTW 30013 was ±400 V, while for IBA CC13, Exradin A1SL, and Exradin A11 was ±300 V.

The determination of position cylindrical or parallel‐plate chamber in the phantom depends on reference depth. The reference depth $z_{\mathrm{ref}}$ for each chamber is determined using the equation $z_{\mathrm{ref}}=0.6R_{50}-0.1\;\left(\mathrm{cm}\right)$, so that $z_{\mathrm{ref}}$ is highly dependent on $R_{50}$. However, there are differences in positioning of the chamber when using modified calibration and TRS‐398. This is explained in more detail in Sections 2.2.1 and 2.2.2. Raw ionization chamber readings are corrected using:

(1) $$M=M_{\mathrm{raw}}P_{\mathrm{TP}}P_{\mathrm{ion}}P_{\mathrm{pol}}P_{\mathrm{elec}}P_{\mathrm{leak}}P_{\mathrm{rp}}$$

where $P_{\mathrm{TP}}$ is the correction to standard environmental conditions of temperature and pressure, $P_{\mathrm{ion}}$ is the ion recombination correction factor, $P_{\mathrm{pol}}$ is the correction for polarity effects, $P_{\mathrm{elec}}$ is the electrometer correction factor, $P_{\mathrm{leak}}$ is the leakage correction factor, and $P_{\mathrm{rp}}$ is the radial profile correction factor to correct for beam non‐uniformity over the chambers central volume. The leakage and radial profile correction factors contribute ≤0.05% and ≤0.15%, respectively. Therefore, these two factors can be neglected in the calculation.^12^

### 2.2 Determination of absorbed dose

The output calibration of dose per monitor unit (*D*/MU) will be obtained through two calculation procedures.

#### 2.2.1 TRS‐398 protocol

According to TRS‐398 protocol, the cylindrical ionization chamber is positioned at 0.5$r_{\mathrm{cyl}}$ deeper than reference depth $z_{\mathrm{ref}}$, while the parallel‐plate chamber is positioned at the reference depth. PTW 30013, IBA CC13, and Exradin A1Sl were used in beams with energies 10, 12, and 15 MeV, while Exradin A11 was used in beams with all energies. The absorbed dose to water is calculated using:

(2) $$D_{w,Q_{o}}=\;M_{Q_{o}}\;N_{D,w,Q_{o}}k_{Q,Q_{o}\;}$$

Data for $k_{Q,Q_{o}\;}$ for cylindrical and parallel‐plate chambers are obtained from Table 7.III in document TRS‐398. Data for the chamber are most similar to the Exradin A1SL, IBA CC13, and Exradin A11, which are Exradin A1, Wellhofer IC‐10, and Exradin P11, respectively.^12^

#### 2.2.2 Modified calibration

Cylindrical chambers are placed with their central axis at $z_{\mathrm{ref}}$. On the other hand, the parallel‐plate chamber is positioned with its effective point of measurement (EPOM) at $z_{\mathrm{ref}}$. For the Exradin A11, measurements performed with EPOM taken to be 1.77 mm (recommended by Muir and Rogers^13^). These chambers are used in beams with all energies. The absorbed dose to water is calculated using the proposed formula^12^ with:

(3) $$D_{w,Q\left(z_{\mathrm{ref}}\right)\;}=\;Mk_{Q}^{\prime }k_{Q,\mathrm{ecal}}N_{D,w}^{\mathrm{Co}}$$

where $k_{Q}^{\prime }$ is a beam quality conversion factor (which do not require an explicit $P_{\mathrm{gr}}^{Q}$ but include gradient effects by definition^12^). The $k_{Q,\mathrm{ecal}}$ factor is fixed for a given chamber type and is simply $k_{R_{50}}$ for an electron beam quality $Q_{\mathrm{ecal}}$ with $R_{50}$ = 7.5 cm.

The determination of $k_{Q}^{\prime }\;$for cylindrical and parallel‐plate chambers are based on previous Monte Carlo calculation.^13^ The determination of $k_{Q}^{\prime }$ for cylindrical chamber is provided by the coefficient of the power fitting parameter (a,b,c) into Equation (4), while for parallel‐plate chamber is provided by the coefficient of the exponential fitting parameter (a,b,c) into Equation (5). The power fitting parameter and exponential fitting parameter of each chamber are shown in Tables 1 and 2. The $R_{50}\;$data for each energy were obtained from the relative measurement results of our institution and presented in Table 3.

(4) $$k_{Q}^{\prime }=\;a+b\;\times \;R_{50}^{-c}$$

(5) $$k_{Q}^{\prime }=\;a+b\;\times \;e^{-R_{50}/c}$$

**TABLE 1:** Power fitting parameters for cylindrical chamber for $k_{Q}^{{}^{\prime }}$ as a function of $R_{50}$ ^12^

		Power fitting parameter			
Manufacturer	Chambers	*a*	*b*	*c*	RMSD (%)
PTW	30013	0.978	0.112	0.816	0.15
IBA	CC13	0.926	0.129	0.279	0.10
Exradin	A1Sl	0.205	0.854	0.036	0.13

*Note*: Cylindrical chambers are positioned with their central axes at $d_{\mathrm{ref}}$ (no effective point of measurement).

Abbreviation: RMSD, root mean square deviation.

**TABLE 2:** Exponential fitting parameters for parallel‐plate chamber for$\;k_{Q}^{{}^{\prime }}$ as a function of $R_{50}$ ^12^

		Exponential fitting parameter			
Manufacturer	Chamber	*a*	*b*	*c*	RMSD (%)
Exradin	A11	0.992	0.114	2.864	0.13

Abbreviation: RMSD, root mean square deviation.

**TABLE 3:** Percentage depth dose (PDD) at $(z_{\mathrm{ref}}$) from Elekta Synergy Platform and Elekta Versa HD linear accelerators

Linear accelerator	$R_{50}\;$(cm)	$z_{\mathrm{ref}}\;$(cm)	PDD $\left(z_{\mathrm{ref}}\right)$
Elekta Synergy Platform	2.37	1.320	99.172
	3.17	1.802	98.810
	3.84	2.204	99.854
	4.68	2.708	99.672
	5.76	3.356	98.684
Elekta Versa HD	2.48	1.390	99.756
	3.23	1.839	99.606
	4.00	2.301	99.616
	4.75	2.750	99.760
	5.96	3.473	98.752

### 2.3 Analysis

The results of the absorbed dose to water for electron beam at $z_{\mathrm{ref}}$ are presented in the form of dose per monitor unit (cGy//MU) at a maximum depth of $z_{\max}$ using the following equation:

(6) $$D_{w,Q}\left(z_{\max}\right)=\frac{100\;\times D_{w,Q}\left(z_{\mathrm{ref}}\right)}{\mathrm{PD}D_{\left(z_{\mathrm{ref}}\right)}}$$

$\mathrm{PD}D_{\left(z_{\mathrm{ref}}\right)}$ is a relative measurement result obtained from our institution and presented in Table 3.

The results of absorbed dose to water for electron beam at the maximum depth $\left(z_{\max}\right)$ according to the modified calibration method were compared with TRS‐398. Based on these results will be obtained the ratio of absorbed dose to water that expressed in $D_{w}\;$and discrepancy value between two methods for each chamber. Both parameters were analyzed based on the dose tolerance limits established by TRS‐398 protocol, which is ±2%.^3^ The dose ratio between the modified calibration and the TRS‐398 of each chamber was then analyzed.

## 3 RESULTS

### 3.1 The beam quality conversion factor $k_{Q,Q_{o}}\;$calculation for ionization chambers by TRS‐398 protocol

The determination of beam quality conversion factor $k_{Q,Q_{o}}$ in TRS‐398 protocol is done by interpolating data in Table 7.III (document of IAEA).The results of $k_{Q,Q_{o}}$ factor for each chamber of the two types of linear accelerators using TRS‐398 are shown in Table 4. Based on Table 4, the results of $k_{Q,Q_{o}}$ factor at 6 and 8 MeV for both types of linear accelerators can be interpolated on the Exradin A11 only. This is different for the other three types of chambers, where the $k_{Q,Q_{o}}$ factor of PTW 30013, IBA CC13, and Exradin A1SL cannot be determined. At 10, 12, and 15 MeV, the four chambers have $k_{Q,Q_{o}}\;$factor that varies depending on $R_{50}$. The $k_{Q,Q_{o}}\;$factor of PTW 30013, IBA CC13, and Exradin A1Sl between the two linear accelerators gave almost the same results. Only at 15 MeV, the $k_{Q,Q_{o}}$ factors of PTW 30013 and IBA CC13 between the two linear accelerators give different $k_{Q,Q_{o}}$ factors. In contrast to the three previous types of chambers, the $k_{Q,Q_{o}}$ factor of Exradin A11 between the two linear accelerators tends to give different results. Overall, the $k_{Q,Q_{o}}$ factor obtained from each chamber using the TRS‐398 protocol is less than 1 with a range of 0.897–0.932.

**TABLE 4:** The results of beam quality factor $k_{Q,Q_{o}}$ calculation for each chamber by Technical Report Series (TRS)‐398 protocol in this work

			$k_{Q,Q_{o}}$			
Linear accelerator	Energy (MeV)	$R_{50}$ (cm)	PTW 30013	IBA CC13	Exradin A1Sl	Exradin A11
Elekta Synergy Platform	6	2.37	–	–	–	0.932
	8	3.17	–	–	–	0.921
	10	3.84	0.911	0.920	0.914	0.915
	12	4.68	0.908	0.918	0.913	0.907
	15	5.76	0.905	0.914	0.912	0.899
Elekta Versa HD	6	2.484	–	–	–	0.930
	8	3.231	–	–	–	0.921
	10	4.002	0.911	0.920	0.914	0.913
	12	4.750	0.908	0.918	0.913	0.906
	15	5.955	0.904	0.913	0.912	0.897

### 3.2 The results of beam quality conversion factor $k_{Q}^{\prime }\;$calculation for ionization chambers by modified calibration

The determination of beam quality of conversion factor between modified calibration and TRS‐398 protocol is different. The determination of $k_{Q}^{\prime }\;$factor in this method is based on the previous work.^13^ Table 5 shows the results of $k_{Q}^{\prime }$ factor for each chamber using a modified calibration method. The $k_{Q}^{\prime }\;$factor of PTW 30013, IBA CC13, and Exradin A1Sl, which is a cylindrical chamber, can be determined in all energy beams. There are different results of the $k_{Q}^{\prime }$ factor for four types of chambers between the two linear accelerators. Overall, the $k_{Q}^{\prime }$ factor obtained from each chamber is more than 1 in the range of 1.004–1.042.

**TABLE 5:** The results of beam quality factor $k_{Q}^{{}^{\prime }}$ calculation for each chamber by modified calibration in this work

			$k_{Q}^{{}^{\prime }}$			
Linear accelerator	Energy (MeV)	$R_{50}$ (cm)	PTW 30013	IBA CC13	Exradin A1Sl	Exradin A11
Elekta Synergy Platform	6	2.37	1.033	1.027	1.033	1.042
	8	3.17	1.022	1.019	1.024	1.030
	10	3.84	1.015	1.015	1.019	1.022
	12	4.68	1.010	1.010	1.013	1.014
	15	5.760	1.005	1.005	1.007	1.007
Elekta Versa HD	6	2.484	1.031	1.026	1.031	1.040
	8	3.231	1.021	1.019	1.024	1.029
	10	4.002	1.014	1.014	1.017	1.020
	12	4.750	1.009	1.010	1.012	1.014
	15	5.955	1.004	1.004	1.006	1.006

**FIGURE 1:**
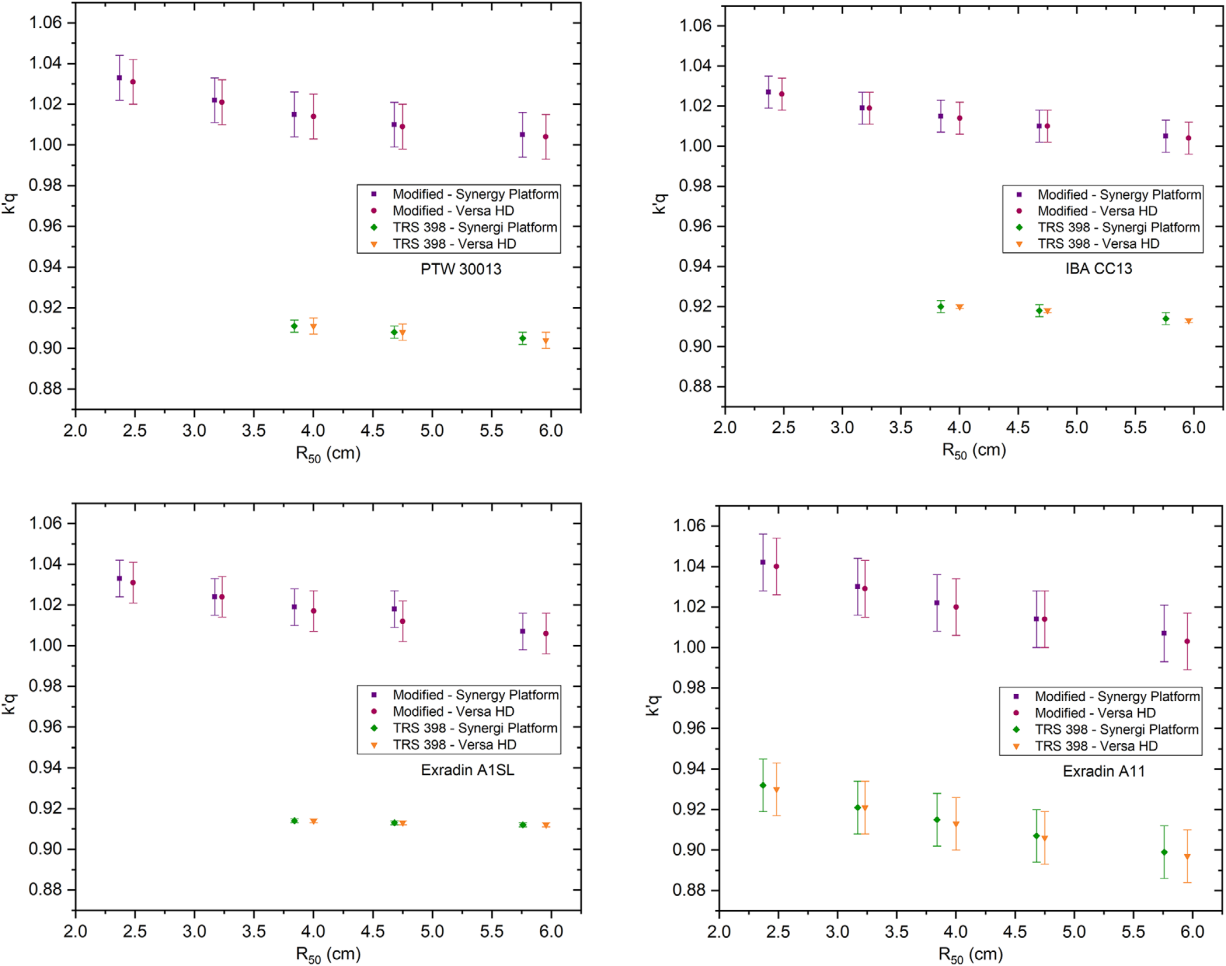
The results of the beam quality conversion factor $k_{Q}^{{}^{\prime }}$ for each ionization chamber using modified calibration and Technical Report Series (TRS)‐398

The beam quality conversion factors between the two methods from each chamber are shown in Figure 1. The $k_{Q}^{\prime }$ factor and error bar from each chamber are also shown in Figure 1. The graphs of PTW 30013, IBA CC13, and Exradin A1SL for *R*_50_ < 3.5 cm only show the results of $k_{Q}^{\prime }$ factor using modified calibration. Based on Figure 1, the standard deviation for the TRS‐398 method tends to be smaller than the modified calibration method, except for Exradin A11. Exradin A11, a parallel‐plate chamber, has the largest standard deviation compared to the other three chambers, 1.4% for the modified calibration method and 1.3% for the TRS‐398 protocol.

### 3.3 The results of absorbed dose to water for electron beam using modified calibration

**FIGURE 2:**
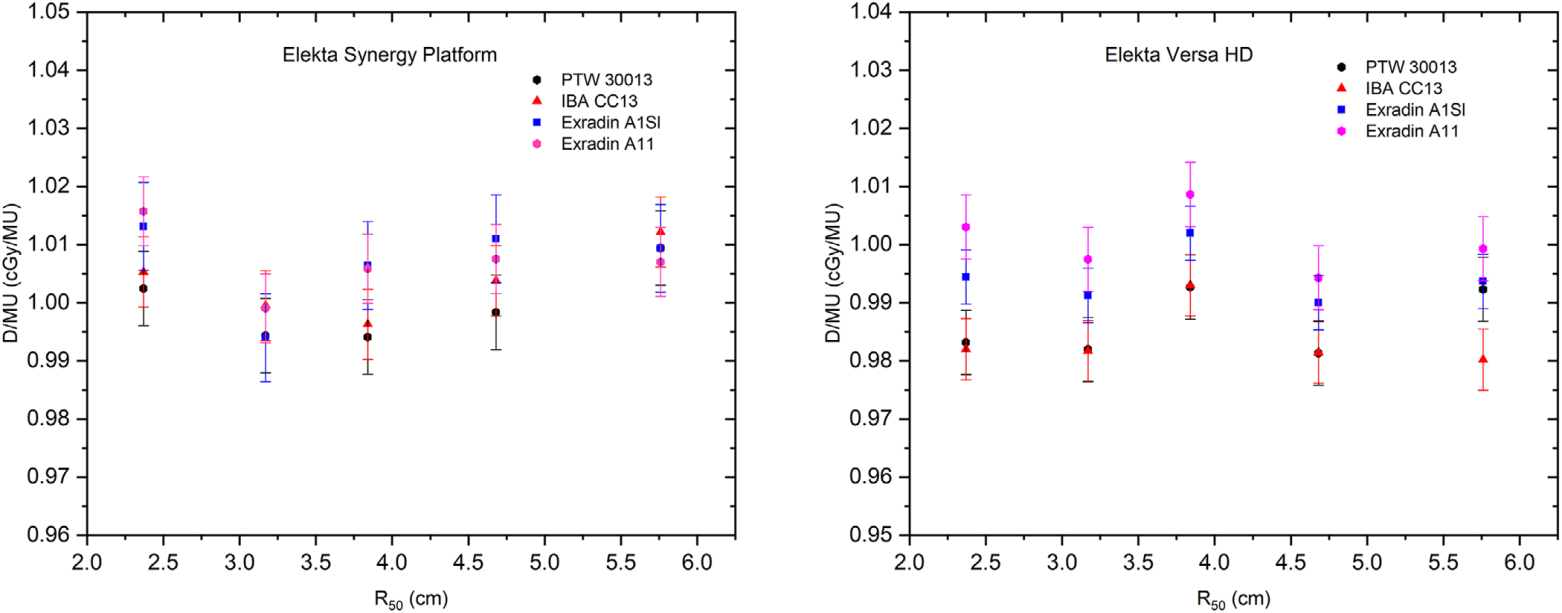
The result of dose per monitor unit (MU) obtained with cylindrical and parallel‐plate chamber using a modified calibration

The results of each chamber's absorbed dose to water using modified calibration are shown in Figure 2. It can be seen that the electron beam calibration results for the two types of linear accelerators are different. The results of the electron beam calibration measurements on the Elekta Versa HD form a pattern, which is different from the Elekta Synergy Platform. Exradin A11 on the Elekta Versa HD gives the highest dose results compared to the other three types of the cylindrical chambers. However, Exradin A1Sl, a cylindrical chamber, gives a dose result almost close to Exradin A11.

The dose comparison and discrepancy of the two methods for cylindrical and parallel‐plate chambers are shown in Tables 6 and 7. $D_{w}$ shows the dose ratio at maximum depth ($z_{\max}$) between modified calibration to TRS‐398. According to Table 6, the dose using the modified calibration of PTW 30013 and IBA CC13 gives smaller results than TRS‐398, which is less than 1, in the range of 0.985–0.999. The discrepancy values from the three types of chambers were varied. PTW 30013 generates a minimum and maximum discrepancy of ‐0.1% and ‐0.6%. CC13 generates minimum and maximum discrepancies of ‐0.4% and ‐1.5%. On the other hand, the results of various dose comparisons of Exradin A1SL, which is also a cylindrical chamber, are shown. The result of dose measurement with Exradin A1SL gave a dose greater than TRS‐398, which is greater than 1 with a value in the range of 1.012–1.019. Exradin A1SL generates the smallest discrepancy of 1.2%.

**TABLE 6:** Ratio of absorbed dose to water of modified calibration and Technical Report Series (TRS)‐398 ($D_{w}$) obtained from three types of cylindrical chamber used in this work

		Elekta Synergy Platform		Elekta Versa HD	
Type of chamber	Energy (MeV)	$D_{w}$	Discrepancy (%)	$D_{w}$	Discrepancy (%)
PTW 30013	6	–	–	–	–
	8	–	–	–	–
	10	0.999	‐0.1	0.994	‐0.6
	12	0.997	‐0.3	0.994	‐0.6
	15	0.999	‐0.1	0.996	‐0.4
IBA CC13	6	–	–	–	–
	8	–	–	–	–
	10	0.988	‐1.2	0.987	‐1.3
	12	0.997	‐0.3	0.988	‐1.2
	15	0.996	‐0.4	0.985	‐1.5
Exradin A1Sl	6	–	–	–	–
	8	–	–	–	–
	10	1.017	1.7	1.018	1.8
	12	1.012	1.2	1.019	1.9
	15	1.012	1.2	1.013	1.3

*Note*: Discrepancy (%) = (absorbed doses for modified calibration – absorbed doses for TRS‐398)/(absorbed doses for TRS‐398 dose).

**TABLE 7:** Ratio of absorbed dose to water of modified calibration and Technical Report Series (TRS)‐398 ($D_{w}$) obtained from parallel‐plate chamber used in this work

		Elekta Synergy Platform		Elekta Versa HD	
Type of chamber	Energy (MeV)	$D_{w}$	Discrepancy (%)	$D_{w}$	Discrepancy (%)
Exradin A11	6	1.013	1.3	1.013	1.3
	8	1.013	1.3	1.013	1.3
	10	1.012	1.2	1.013	1.3
	12	1.014	1.4	1.014	1.4
	15	1.015	1.5	1.016	1.6

*Note*: Discrepancy (%) = (absorbed doses for modified calibration – absorbed doses for TRS‐398)/(absorbed doses for TRS‐398 dose).

Table 7 shows the dose ratios measured with Exradin A11 at energies of 6, 8, and 12 MeV that gave the same results for both linear accelerators. At 6 and 8 MeV, the measured dose ratio was 1.013 with a deviation of 1.3%. The lowest dose discrepancy is 1.2% at 10 MeV from Elekta Synergy Platform, while the highest dose deviation is 1.6% at 15 MeV from Elekta Versa HD. Based on Table 7, the dose calculation with modified calibration for Exradin A11 gives a result greater than TRS‐398 with a value in the range of 1.013–1.016. It is similar to Exradin A1SL cylindrical chamber from the same manufacturer. Based on Tables 6 and 7, the ratio of the average absorbed dose for all energies between the modified calibration and TRS‐398 (*D*_w_) for the cylindrical chamber was 1.002 on the Elekta Synergy Platform and 1.000 on the Versa HD while for the parallel‐plate chamber it was 1.013 on the Elekta Synergy Platform and 1.014 on the Versa HD.

**FIGURE 3:**
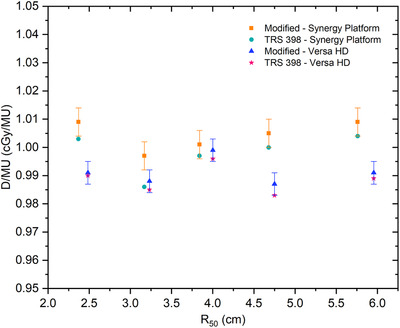
The result of average dose per monitor unit a from different types of chamber according to modified calibration and Technical Report Series (TRS)‐398

The results of the average dose per monitor unit from different types of the chamber for each $R_{50}$ are shown in Figure 3. The error bar shown in the graph shows the standard deviation of the modified calibration method. The result of the average dose per monitor unit at 6 MeV ($R_{50}\;$= 2.37 and 2.48 cm) and 8 MeV ($R_{50}$ = 3.17 and 3.23 cm) with TRS‐398 is the dose contribution from Exradin A11. The error bar of the average dose per monitor unit from different types of chambers on the Elekta Synergy Platform and Elekta Versa HD is 0.5% and 0.4%, respectively.

**FIGURE 4:**
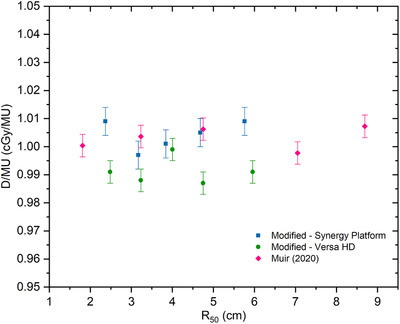
The results of dose per monitor unit calculation from this work compared to the previous work^12^

Figure 4 shows the results of the average dose per unit monitor in this study compared to the previous work.^12^ The error bar in Figure 4 shows the variability of the results obtained with different chambers. As previously explained in Figure 3, the standard deviation of the dose per monitor unit obtained in this study was 0.5% on the Elekta Synergy Platform and 0.4% on the Elekta Versa HD. The result of the standard deviation of the dose per monitor unit by the previous work^12^ was 0.4%. Therefore, it can be seen that the dose per monitor unit using a modified calibration for electron beam in this study gave good results.

## 4 DISCUSSIONS

The determination of $k_{Q,Q_{o}}$ factor with TRS‐398 protocol, which is a function of $R_{50}$, can be known by interpolating the data. The $k_{Q,Q_{o}}$ factor in this protocol at 6 and 8 MeV of the two types of linear accelerators can only be interpolated on Exradin A11, while it cannot be determined for the other three types of chambers. This is because the $k_{Q,Q_{o}}$ factor for PTW 30013, IBA CC13, and Exradin A1Sl for energies below 10 MeV ($R_{50}\leq 4.0$) were not found in Table 7.III (IAEA TRS‐398 document).^3^ This is in line with the TRS‐398, the protocol which does not recommend the use of cylindrical chambers below 10 MeV. At 10, 12, and 15 MeV, the four types of chambers have $k_{Q,Q_{o}}\;$that vary depending on $R_{50}$.

The average results of $k_{Q}^{\prime }$ by modified calibration are greater than TRS‐398. This is explained by recent work^12^ where the determination of the electron beam quality conversion factor has implicitly included the gradient correction factor $P_{\mathrm{gr}}^{Q}$ into $k_{Q}^{\prime }$. The beam quality conversion factor $k_{Q}^{\prime }$ is determined by using a complete Monte Carlo calculation.^13^ It has combined the detailed information about the chamber to reflect the actual chamber geometry. This is different from the results of using TRS‐398 calculation, where the $k_{Q,Q_{o}}$ factor is based on a semi‐analytical approach that does not consider all the details of the chamber geometry.^14^

The difference of beam quality factor generated by the two types of linear accelerators is due to the difference of $R_{50}$. Based on Table 3, it is known that the $R_{50}$ obtained by our institution for the Elekta Synergy Platform is lower than that of Elekta Versa HD. Since the difference of$\;R_{50}$ between the two linear accelerators is not too big, the difference beam quality factor is also less.

Since the calculation of absorbed dose for the electron beam at low energy with cylindrical chambers cannot be determined using TRS‐398, it is not possible to determine the ratio of the modified calibration and TRS‐398 at 6 and 8 MeV for incorporation into Table 6. This was explained before on the $k_{Q,Q_{o}}$ calculation using TRS‐398. Thus, the calculation of the absorbed dose for electron beam at low energy cannot be determined with TRS‐398. However, a comparison of the modified calibration using cylindrical chamber with TRS‐398 using a parallel‐plate chamber can be made. Three types of cylindrical chambers produce a minimum and maximum dose deviation of ‐0.1% and 1.9%. These results show that dose calculations using a cylindrical chamber for electron beam calibration purposes give acceptable results. This is because IAEA recommends calculating the radiation dose with a tolerance limit of ±2%.^3^ Based on previous work^15^ the use of a cylindrical chamber is due to energy‐independent electron beam dosimetry. In another previous work,^11^ the variability of perturbation correction factor on the Farmer‐type cylindrical chamber was examined and gave a result of less than 0.4%. Therefore, this work suggested that the use of a cylindrical chamber as electron beam dosimetry is suitable at all energies.

The dose difference between the two protocols generated by the three types of cylindrical chambers is caused by $k_{Q}^{\prime }$. The result of $k_{Q}^{\prime }$ calculation for Exradin A1SL is higher than the other two cylindrical chambers, which have a larger volume. This is due to the substitute correction factor's small effect on the Exradin A1SL.^16^ This is in line with the previous work,^17^ that computes the fluence correction factor as a function of the radius cavity and $R_{50}$. They explained fewer different results for the chamber with smaller radius cavity.

The discrepancy of measured dose between the two methods is also due to the displacement correction factor or $P_{\mathrm{dis}}$ of each chamber. This happens because in modified calibration, in which the reference conditions generally follow the AAPM TG‐51 protocol, the position of the cylindrical chamber is set to the reference depth ($z_{\mathrm{ref}})$, where $P_{\mathrm{dis}}$ is not required. In contrast to the TRS‐398 protocol, in which the cylindrical chamber position at the EPOM, is 0.5$r_{\mathrm{cyl}}$ deeper than reference depth ($z_{\mathrm{ref}}$). The same applies to the parallel‐plate chamber, where the positioning of the parallel‐plate chamber is optimally shifted when modified calibration is applied. The positioning of the parallel‐plate chamber is at the reference depth when TRS‐398 is applied.^3^

The resulting dose ratios determined in this work were lower than in previous work. Based on the percentage difference values, it can be shown that measurements with Elekta Synergy Platform compared to Elekta Versa HD are closer to the results of previous work. The average dose per monitor unit with different chambers represents small variability between our result and Muir's result. This shows that using a modified calibration method to calculate the electron absorption dose from different chamber types provides good consistency of results across all energy beams.

## 5 CONCLUSION

In this work, we have succeeded in implementing the modified calibration of electron beam based on TRS‐398 by applying a cylindrical chamber in all energy beams and using the updated $k_{Q}$ factor. The average ratio of absorbed dose between modified calibration and TRS‐398 ($D_{w}$) gave a smaller value for PTW 30013 and IBA CC13, while the larger value was obtained for Exradin A1SL and Exradin A11. The ratio of the absorbed dose to water between the modified calibration to TRS‐398 (*D*_w_) for the cylindrical chamber was 1.002 on the Elekta Synergy Platform and 1.000 on the Versa HD while for the parallel‐plate chamber it was 1.013 on the Elekta Synergy Platform and 1.014 on the Versa HD. Based on these results, both the cylindrical and parallel‐plate chambers are still within the tolerance limit allowed by the TRS‐398 protocol, which is ±2%. The standard deviation of the average dose per monitor unit from different types of chambers on the Elekta Synergy Platform and Elekta Versa HD is 0.5% and 0.4%, respectively. This result is in line with standard deviation which is provided by the previous work.^12^ Therefore, it can be seen that the dose per monitor unit using a modified calibration for electron beam in this study gave good results.

## AUTHOR CONTRIBUTIONS

Supriyanto A. Pawiro was responsible in research design,data collection, data analysis, and manuscript writing. Dwi A. Mahfirotin was responsible in data collection, data analysis and manuscript writing. Muhamad I. Assegab contributed in data collection. Wahyu E. Wibowo conributed in data collection.

## CONFLICT OF INTEREST

The authors declare no potential conflict of interest.
